# Contribution of serotonin receptor subtypes to hallucinogenic activity of 25I-NBOMe and to its effect on neurotransmission

**DOI:** 10.1007/s43440-020-00181-4

**Published:** 2020-11-10

**Authors:** Monika Herian, Adam Wojtas, Małgorzata Katarzyna Sobocińska, Mateusz Skawski, Alejandro González-Marín, Krystyna Gołembiowska

**Affiliations:** Department of Pharmacology, Maj Institute of Pharmacology, Polish Academy of Sciences, 12 Smętna, 31-343 Kraków, Poland

**Keywords:** 25I-NBOMe, Hallucinogenic activity, Neurotransmission, 5-HT_2A/2C/1A_ receptor antagonists

## Abstract

**Background:**

4-Iodo-2,5-dimethoxy-*N*-(2-methoxybenzyl)phenethylamine (25I-NBOMe) is a potent serotonin (5-HT) receptor agonist with hallucinogenic properties. The aim of our research was to examine the role of the 5-HT_2A_, 5-HT_2C_ and 5-HT_1A_ serotonin receptor subtypes in 25I-NBOMe hallucinogenic activity and its effect on dopamine (DA), 5-HT and glutamate release in the rat frontal cortex.

**Methods:**

Hallucinogenic activity was investigated using the wet dog shake (WDS) test. The release of DA, 5-HT and glutamate in the rat frontal cortex was studied using a microdialysis in freely moving rats. Neurotransmitter levels were analyzed by HPLC with electrochemical detection. The selective antagonists of the 5-HT_2A_, 5-HT_2C_ and 5-HT_1A_ serotonin receptor subtypes: M100907, SB242084 and WAY100635, respectively were applied through a microdialysis probe.

**Results:**

The WDS response to 25I-NBOMe (1 and 3 mg/kg) was significantly reduced by local administration of M100907 and SB242084 (100 nM). The 25I-NBOMe-induced increase in glutamate, DA and 5-HT release was inhibited by M100907 and SB242084. WAY100635 had no effect on 25I-NBOMe-induced WDS and glutamate release, while it decreased DA and 5-HT release from cortical neuronal terminals.

**Conclusion:**

The obtained results suggest that 5-HT_2A_ and 5-HT_2C_ receptors play a role in 25I-NBOMe-induced hallucinogenic activity and in glutamate, DA and 5-HT release in the rat frontal cortex as their respective antagonists attenuated the effect of this hallucinogen. The disinhibition of GABA cells by the 5-HT_1A_ receptor antagonist seems to underlie the mechanism of decreased DA and 5-HT release from neuronal terminals in the frontal cortex.

**Electronic supplementary material:**

The online version of this article (10.1007/s43440-020-00181-4) contains supplementary material, which is available to authorized users.

## Introduction

4-Iodo-2,5-dimethoxy-*N*-(2-methoxybenzyl)phenethylamine (25I-NBOMe) is a synthetic * N*-methoxybenzyl derivative of 2C-I (4-iodo-2,5-dimethoxyphenethylamine), a compound from the phenylalkylamine family and a potent 5-HT receptor agonist. 25I-NBOMe exhibits high in vitro binding affinities for 5-HT_2A_, 5-HT_2C_ and 5-HT_1A_ receptors (*Ki* = 0.6, 4.6 and 1800 nM, respectively) [1].

Serotonin receptors are the main target for the action of classical hallucinogens. Serotonergic hallucinogens that act via these receptors include indoleamines and phenylalkylamines which display a high binding affinity for serotonin 5-HT_2A_, 5-HT_2C_ and 5-HT_1A_ receptor subtypes [2]. Serotonin-2A receptors are expressed mainly on the apical dendrites of pyramidal cells in layer V of the cerebral cortex [3] with a minor localization to GABAergic interneurons [4, 5]. Their activation leads to enhancement of cortical glutamate release, which is a common mechanism of action of hallucinogens [6–9]. Serotonin-2C receptors expressed on GABAergic cells in the deep layers of the prefrontal cortex may exert an inhibitory tone on pyramidal neurons by GABA release [10, 11], whereas 5-HT_1A_ as postsynaptic heteroreceptors are localized mainly on pyramidal neurons and on GABAergic interneurons [12].

Many hallucinogenic drugs that act as 5-HT_2A_ receptor agonists evoke a rhythmic paroxysmal rotational head movement in rodents, known in mice as head twitch response (HTR) and also referred to as a wet dog shakes (WDS) in rats [13]. It was shown that 5-HT_1A_ and 5-HT_2C_ receptor activation negatively modulated the 5-HT_2A_-mediated effect on head twitch episodes in rodents [14]. It was proven that higher doses of some hallucinogenic agents, apart from the activation of 5-HT_2A_ receptors, might also cause activation of 5-HT_2C_ receptors which opposed the effect of 5-HT_2A_ receptor stimulation [15]. For instance, 2,5-dimethoxy-4-iodoamphetamine (DOI)-elicited head twitch behavior was inhibited by competing 5-HT_2C_ agonist activity [16]. However, there is evidence that stimulation of both 5-HT_2A_ and 5-HT_2C_ receptors may be required for hallucinogenic activity [2]. Furthermore, 5-HT_1A_ receptors co-expressing with 5-HT_2A_ receptors in cortical pyramidal neurons often show the opposite effect on common signaling pathways [17], which results in the inhibition of the functional effects mediated by 5-HT_2A_ receptors and attenuation of HTR episodes evoked by tryptamine-like hallucinogens [14].

There are studies confirming that 25I-NBOMe, through its agonist activity at the 5-HT_2A_ receptor, produces dose-dependent increases in HTR/WDS episodes as well as back muscle contractions (BMC) in rodents [6, 13, 18, 19]. Pretreatment with the selective 5-HT_2A_ antagonist M100907 caused a significant reduction in WDS and BMC in rats, evoked by 25I-NBOMe [18]. Similarly, HTR elicited by 25I-NBOMe in mice was also blocked by M100907 [19]. Moreover, the increase in glutamate level induced by intracortical injections of DOI was blocked by M100907 [9]. 25CN-NBOH, another representative of the phenethylamine series of hallucinogens, evoked HTR episodes that were blocked by pretreatment with the 5-HT_2A_ antagonist ketanserin [20]. Instead, the 5-HT_2C_ receptor antagonist SB242084 produced a slight increase in the number of head twitches elicited by 25CN-NBOH [20]. On the other hand, there is a study indicating that DOI-induced HTR episodes were reduced but not eliminated after pretreatment of mice with 5-HT_2C_ receptor antagonists (SB206553 or SB242084) [21].

Our previous study demonstrated that 25I-NBOMe exerted a potent effect on cortical glutamate, DA and 5-HT levels and induced hallucinogenic activity [6]. There are no data on the contribution of cortical 5-HT receptor blockade to the effect of 25I-NBOMe on brain neurotransmission correlated with behavioral response. Therefore, the aim of this study was to assess the role of serotonin 5-HT_2A_, 5-HT_2C_ and 5-HT_1A_ receptors in the changes induced by 25I-NBOMe using selective 5-HT receptor antagonists (M100907, SB242084 and WAY100635). We examined the impact of 5-HT receptor antagonists, administered locally into the rat frontal cortex, on the changes in extracellular cortical levels of glutamate, DA and 5-HT induced by 25I-NBOMe. Shaking behavior was also tested as an indicator of hallucinogenic activity.

## Materials and methods

### Animals

All experiments were performed on male Wistar-Han rats (Charles River, Sulzfeld, Germany) weighting 280–320 g. The animals were initially acclimatized and housed in groups of 5 each in temperature (23 ± 1 °C) and humidity (55 ± 10%) controlled rooms under a 12 h light/dark cycle (light was turned on at 6 a.m.), with free access to tap water and standard laboratory food (VRF 1, Special Diets Services, Witham, UK). The experiments were conducted in accordance with the European regulations for animal experimentation (EU Directive 2010/63/EU on the protection of animals used for scientific purposes). The experimental protocols were approved by the Local Ethics Commission for Experimentation on Animals (Permit number: 187/2017). This article does not contain any studies with human participants by any of the authors.

### Drugs and reagents

4-Iodo-2,5-dimethoxy-*N*-(2-methoxybenzyl)phenethylamine hydrochloride (25I-NBOMe) was purchased from Chiron AS (Trondheim, Norway). All chemicals used for high-performance liquid chromatography (HPLC) were obtained from Merck (Warszawa, Poland), O-phthalaldehyde (OPA) was from Sigma-Aldrich. Ketamine hydrochloride and xylazine hydrochloride were from Biowet (Pulawy, Poland). M100907, SB242084 dihydrochloride hydrate and WAY100635 maleate salt were from Sigma-Aldrich (Poznan, Poland).

### Drug administration

**Scheme 1:**
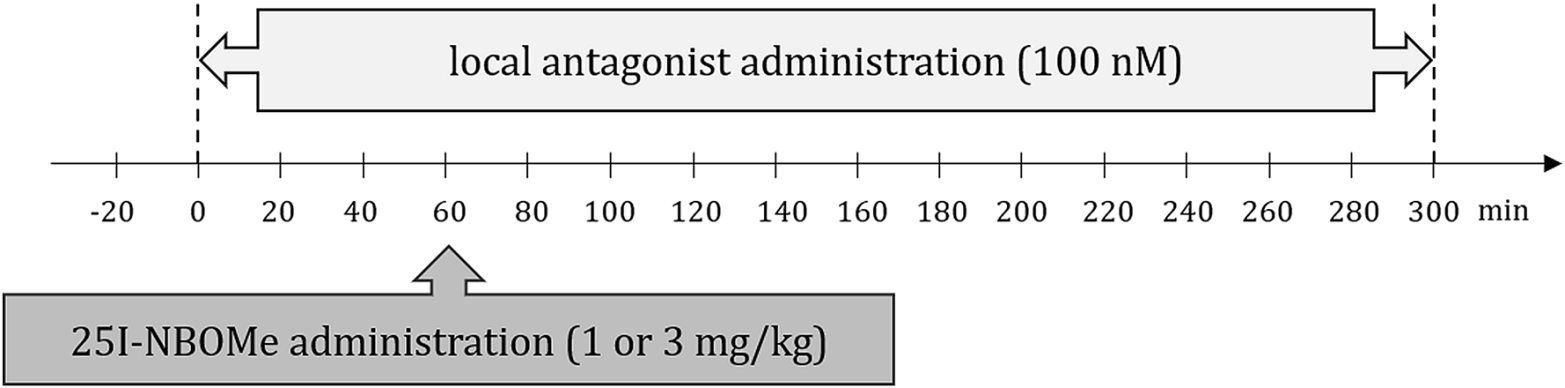
The drug administration schedule is shown on the graph above

Solutions of the antagonists were prepared by dissolving M100907, SB242084 and WAY100635 in artificial cerebrospinal fluid and were administered locally via reverse microdialysis. The value of 100 nM was derived from the concentration–response curve of 5-HT_2A/2C/1A_ receptor antagonists determined in preliminary experiments. The animals received single subcutaneous (sc) injections of 25I-NBOMe dissolved in 0.9% NaCl at a dose of 1 or 3 mg/kg. These doses were established according to our previous study [6]. The control group was treated with antagonists and 0.9% NaCl solution in the same way. The drug administration schedule is shown on the graph in Scheme 1.

### Brain microdialysis

**Fig. 1:**
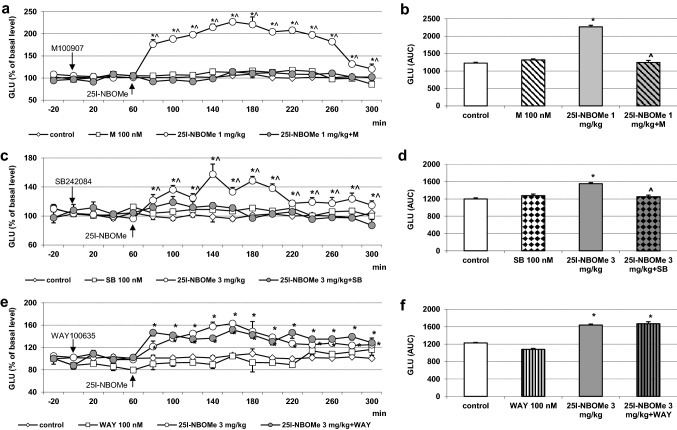
The time-course (**a**, **c**, **e**) and total (**b**, **d**, **f**) effect of 25I-NBOMe and M100907, SB242084 and WAY100635, respectively on extracellular levels of glutamate (GLU) in the rat frontal cortex. The total effect was calculated as an area under the concentration–time curve (AUC) and expressed as the percent of control. Values are the mean ± standard error of the mean (SEM), *n* = 5–6 per experimental group. The administration of drugs is indicated with arrows. *p < 0.01 vs. control group; ^*p* < 0.01 25I-NBOMe vs. 25I-NBOMe + M100907, 25I-NBOMe + SB242084 or 25I-NBOMe + WAY100635 groups (two-way repeated measures ANOVA or two-way ANOVA where appropriate and Tukey’s post hoc test)

Animals were anesthetized intramuscularly with ketamine (75 mg/kg) and xylazine (10 mg/kg), placed in a stereotaxic apparatus (David Kopf Instruments, Tujunga, USA) and guide cannulas were implanted in the rat frontal cortex. The animals were allowed to recover for 3 days and then microdialysis probes (MAB 4.15.4.Cu AgnTho’s AB, Lindingö, Sweden) were inserted according to coordinates (mm) AP + 2.7, L + 0.8, V − 6.5 from the dura [22] and probe inlets were connected to a syringe pump (BAS, West Lafayette, IN, USA). The artificial cerebrospinal fluid (aCSF) composed of NaCl (147 mM), KCl (4 mM), CaCl_2_ (2.2 mM) and MgCl_2_ (1.0 mM) was delivered at a flow rate of 2 μL/min for 2 h of washout period. After collection of four 20-min basal dialysate samples, appropriate 5-HT receptor subtype antagonists were administered through microdialysis probes. Following 1 h of drug infusion, rats were injected with appropriate 25I-NBOMe doses and sample collection continued for 240 min. At the end of the experiment, the animals were sacrificed and their brains were examined histologically to ensure that probes were placed correctly (Fig. 1S, Supplementary data).

### Analytical procedure

DA and 5-HT levels in dialysate fractions were analyzed by HPLC with amperometric detection using an Ultimate 3000 System (Dionex, Sunnyvale, CA, USA), electrochemical detector Coulochem III (model 5300; ESA, Chelmsford, MA, USA) with a 5020 guard cell, 5040 amperometric cell using a Hypersil Gold C18 column (3 μm, 100 × 3 mm; Thermo Fisher Scientific, Waltham, MA, USA) controlled by Chromeleon v.6.80 (Dionex, Sunnyvale, CA, USA) software. The mobile phase consisted of 0.1 M potassium phosphate buffer adjusted to pH 3.8, 0.5 mM Na_2_EDTA, 100 mg/L 1-octanesulfonic acid sodium salt, and 2% methanol. The flow rate was 0.6 mL/min. The applied potential of a guard cell was 600 mV and amperometric cell was 300 mV with a sensitivity set at 10 nA/V. The limit of detection of DA and 5-HT in dialysates was 0.002 pg/10 μL for DA and 0.01 pg/10 μL for 5-HT.

Glutamate concentration in the extracellular fluid was measured electrochemically after derivatization with OPA/sulfite reagent to form an isoindole-sulfonate derivative [23] using an Ultimate 3000 pump (Dionex, Sunnyvale, CA, USA), LC-4B amperometric detector with a cross-flow detector cell (BAS) and HR-80 column (3 μm, 80 × 4.6 mm; ESA Inc, Chelmsford, MA, USA) controlled by Chromax 2005 (Pol-Lab, Warszawa, Poland) software. The mobile phase was composed of 100 mM monosodium orthophosphate at pH 4.6 and 4% methanol. The flow rate during analysis was set to 1 mL/min and the applied potential of a 3-mm glassy carbon electrode was set at + 600 mV at a sensitivity of 5 nA/V. Glutamate-derivative peak was compared with the respective standard and the data were processed using Chromax 2005 (Pol-Lab, Poland) software. The limit of detection of glutamate in dialysates was 0.03 ng/10 μL.

### Wet dog shake test

The wet dog shake (WDS) test was carried out after administration of drugs according to the procedure described by Nagayama and Lu [24] adapted to the experimental design. Shaking behavior was defined as a rapid, side-to-side rotational movement of the head with propagation to neck, shoulders and trunk, and each such episode was counted. Directly after administration of drugs, rats were observed by two independent and experienced experimenters who were blind to the treatments. The number of shakes was counted and totaled from 12 observation periods. Results were expressed as an average of sum values from 12 observation periods.

### Data analysis

Drug effects on DA, 5-HT and glutamate release in the rat frontal cortex were analyzed with two-way repeated measures ANOVA on normalized responses followed by Tukey’s post hoc test. All obtained data were presented as a percent of the basal level assumed to be 100%. The total effects expressed as an area under the curve (AUC) representing the time-course of drug effects were analyzed with two-way ANOVA. The results from the WDS test were analyzed using two-way ANOVA followed by Tukey’s post hoc test. The differences were considered significant if *p* < 0.05. All statistical analyses were carried out using STATISTICA v.10 StatSoft Inc. 1984–2011 (San Francisco, CA, USA).

## Results

### The effect of M100907, SB242084 and WAY100635 on 25I-NBOMe-induced increase in glutamate levels in the rat frontal cortex

The extracellular levels of glutamate were not affected by administration through a dialysis probe, of the 5-HT_2A_ receptor antagonist M100907, 5-HT_2C_ receptor antagonist SB242084 and 5-HT_1A_ receptor antagonist WAY100635 at a concentration of 100 nM (Fig. 1a, c, e). 25I-NBOMe significantly increased extracellular glutamate levels (*p* < 0.01) in the frontal cortex. The dose of 1 mg/kg was more potent than the dose of 3 mg/kg (Fig. 1a, c, e). M100907 and SB242084 reduced the 25I-NBOMe-induced increase in the extracellular glutamate level in the frontal cortex to the level of control group (Fig. 1a, c). The 25I-NBOMe-induced increase in the extracellular glutamate level was not changed by WAY100635 (Fig. 1e). Two-way repeated measures ANOVA showed a significant effect of treatment with 25I-NBOMe and M100907 (*F*_3,18_ = 1987, *p* < 0.0001), sampling period (*F*_11,198_ = 55, *p* < 0.0001), and time × treatment interaction (*F*_33,198_ = 29, *p* < 0.0001). There was also an effect of treatment with 25I-NBOMe and SB242084 (*F*_3,18_ = 133, *p* < 0.0001) and time (*F*_11,198_ = 5.9, *p* < 0.0001), and time × treatment interaction (*F*_33,198_ = 5.9, *p* < 0.0001). The effect of treatment with 25I-NBOMe and WAY100635 was significant (*F*_3,18_ = 160, *p* < 0.0001), there was also an effect of time (*F*_11,198_ = 15, *p* < 0.0001) and time × treatment interaction (*F*_33,198_ = 6.1, *p* < 0.0001). The total effects expressed as AUC shown in Fig. 1b, d, f reflect the responses to 25I-NBOMe and 25I-NBOMe plus antagonists of 5-HT_2A/2C/1A_ receptors with respect to cortical glutamate release presented as time-course curves. Data set of the total effects was subjected to two-way ANOVA. The group factor was significant for 25I-NBOMe in each case: *F*_1,18_ = 1867, *p* < 0.0001 (Fig. 1b); *F*_1,18_ = 127, *p* < 0.0001 (Fig. 1d) and *F*_1,18_ = 470, *p* < 0.0001 (Fig. 1f). The group factor was also significant for M100907 and SB242084: *F*_1,18_ = 1708, *p* < 0.0001; *F*_1,18_ = 91, *p* < 0.0001 (Fig. 1b and 1d, respectively), but not for WAY100635: *F*_1,18_ = 3.43, *p* < 0.08 (Fig. 1f). The group factor was significant for 25I-NBOMe + M100907 and 25I-NBOMe + SB242084: *F*_1,18_ = 1998, *p* < 0.0001; *F*_1,18_ = 154, *p* < 0.0001, respectively (Fig. 1b and 1d), but not for 25I-NBOMe + WAY100635: *F*_1,18_ = 0.08, *p* < 0.79 (Fig. 1f). Post hoc Tukey’s test showed a significant difference between control and 25I-NBOMe (1 and 3 mg/kg) and 25I-NBOMe + WAY100635 groups (*p* < 0.01) (Fig. 1b, d, f) and between 25I-NBOMe + M100907 and 25I-NBOMe + SB242084 in comparison to 25I-NBOMe alone (*p* < 0.01) (Fig. 1b and d).

### The effect of M100907, SB242084 and WAY100635 on 25I-NBOMe-induced increase in DA levels in the rat frontal cortex

**Fig. 2:**
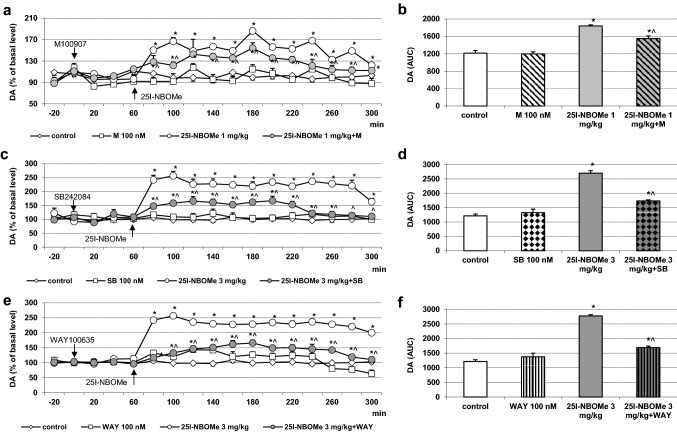
The time-course (**a**, **c**, **e**) and total (**b**, **d**, **f**) effect of 25I-NBOMe and M100907, SB242084 and WAY100635, respectively on extracellular levels of dopamine (DA) in the rat frontal cortex. The total effect was calculated as an area under the concentration–time curve (AUC) and expressed as the percent of control. Values are the mean ± standard error of the mean (SEM), *n* = 5–6 per experimental group. The administration of drugs is indicated with arrows. *p < 0.01 vs. control group; ^*p* < 0.01 25I-NBOMe vs. 25I-NBOMe + M100907, 25I-NBOMe + SB242084 or 25I-NBOMe + WAY100635 groups (two-way repeated measures ANOVA or two-way ANOVA where appropriate and Tukey’s post hoc test)

The extracellular levels of DA were not changed by administration of the 5-HT_2A_, 5-HT_2C_ and 5-HT_1A_ receptor antagonists M100907, SB242084 and WAY100635, respectively, at a concentration of 100 nM through a dialysis probe (Fig. 2a, c, e). The dose of 1 and 3 mg/kg of 25I-NBOMe significantly (*p* < 0.01) increased extracellular levels of DA in the rat frontal cortex (Fig. 2a, c, e, respectively). M100907, SB242084 and WAY100635 suppressed 25I-NBOMe-induced increase in the extracellular DA levels, but the effect of M100907 was weaker than that of SB242084 and WAY100635 (Fig. 2a, c, e). Two-way repeated measures ANOVA showed a significant effect of 25I-NBOMe and M100907 (*F*_3,18_ = 314, *p* < 0.0001), sampling period (*F*_11,198_ = 7.7, *p* < 0.0001), and time × treatment interaction (*F*_33,198_ = 3.4, *p* < 0.0001). The effect of treatment with 25I-NBOMe and SB242084 was also significant (*F*_3,18_ = 647, *p* < 0.0001), there was an effect of sampling period (*F*_11, 198_ = 10.4, *p* <  0.0001) and time × treatment interaction (*F*_33,198_ = 5.7, *p* < 0.0001). There was also an effect of treatment with 25I-NBOMe and WAY100635 (*F*_3,18_ = 586, *p* < 0.0001), sampling period (*F*_11,198_ = 8.1, *p* < 0.0001), and time × treatment interaction (*F*_33,198_ = 5.6, *p* < 0.0001). The total effects expressed as AUC shown in Fig. 2b, d, f reflect the responses to 25I-NBOMe and 25I-NBOMe plus the 5-HT_2A/2C/1A_ receptor antagonists with respect to cortical DA release presented as time-course curves. Data set of the total effects was subjected to two-way ANOVA. The group factor was significant for 25I-NBOMe in each case: *F*_1,18_ = 753, *p* < 0.0001 (Fig. 2b); *F*_1,18_ = 1231, *p* < 0.0001 (Fig. 2d) and *F*_1,18_ = 1358, *p* < 0.0001 (Fig. 2f). The group factor was also significant for M100907, SB242084: *F*_1,18_ = 66, *p* < 0.0001; *F*_1,18_ = 288, *p* < 0.0001 and WAY100635 *F*_1,18_ = 378, *p* < 0.0001 (Fig. 2b, d and f, respectively). The group factor was significant for 25I-NBOMe + M100907 and 25I-NBOMe + SB242084: *F*_1,18_ = 74, *p* < 0.0001; *F*_1,18_ = 299, *p* < 0.0001, respectively and for 25I-NBOMe + WAY100635: *F*_1,18_ = 390, *p* < 0.0001 (Fig. 2b, d and f, respectively). Post hoc Tukey’s test showed significant difference between control and 25I-NBOMe (1 and 3 mg/kg) and 25I-NBOMe + M100907, 25I-NBOMe + SB242084 and 25I-NBOMe + WAY100635 groups (*p* < 0.01), and between 25I-NBOMe + M100907 and 25I-NBOMe + SB242084 and 25I-NBOMe + WAY100635 in comparison with 25I-NBOMe alone (*p* < 0.01) (Fig. 2b, d and f).

### The effect of M100907, SB242084 and WAY100635 on 25I-NBOMe-induced increase in 5-HT levels in the rat frontal cortex

**Fig. 3:**
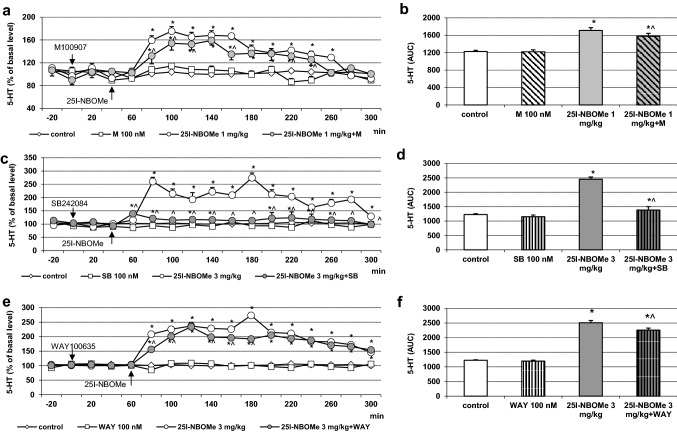
The time-course (**a**, **c**, **e**) and total (**b**, **d**, **f**) effect of 25I-NBOMe and M100907, SB242084 and WAY100635, respectively on extracellular levels of serotonin (5-HT) in the rat frontal cortex. The total effect was calculated as an area under the concentration–time curve (AUC) and expressed as the percent of control. Values are the mean ± standard error of the mean (SEM), *n* = 5–6 per experimental group. The administration of drugs is indicated with arrows. *p < 0.01 vs. control group; ^*p* < 0.01 25I-NBOMe vs. 25I-NBOMe + M100907, 25I-NBOMe + SB242084 or 25I-NBOMe + WAY100635 groups (two-way repeated measures ANOVA or two-way ANOVA where appropriate and Tukey’s post hoc test)

The extracellular levels of 5-HT were not changed by administration of the 5-HT_2A_, 5-HT_2C_ and 5-HT_1A_ receptor antagonists M100907, SB242084 and WAY100635, respectively, at the concentration of 100 nM through a dialysis probe (Fig. 3a, c, e). Both doses of 1 and 3 mg/kg of 25I-NBOMe significantly (*p* < 0.01) increased extracellular levels of 5-HT in the rat frontal cortex (Fig. 3a, c, e, respectively). SB242084 reduced the 25I-NBOMe-induced increase in extracellular 5-HT level nearly to the control values (Fig. 3c), while the effect of M100907 and WAY100635 was weaker but significant (Fig. 3a, e). Two-way repeated measures ANOVA showed a significant effect of treatment with 25I-NBOMe and M100907 on 5-HT release (*F*_3,18_ = 359, *p* < 0.0001), sampling period (*F*_11,198_ = 46, *p* < 0.0001), and time × treatment interaction (*F*_33,198_ = 15.8, *p* < 0.0001). There was also an effect of treatment with 25I-NBOMe and SB242084 (*F*_3,18_ = 544, *p* < 0.0001), sampling period (*F*_11,198_ = 15.0, *p* < 0.0001), and time × treatment interaction (*F*_33,198_ = 12.1, *p* < 0.0001). The effect of treatment with 25I-NBOMe and WAY100635 was significant (*F*_3,18_ = 359, *p* < 0.0001), as were sampling period (*F*_11,198_ = 46, *p* < 0.0001) and the time × treatment interaction (*F*_33,198_ = 15.8, *p* < 0.0001). Data set of the total effects was subjected to two-way ANOVA. The group factor was significant for 25I-NBOMe in each case: *F*_1,18_ = 986, *p* < 0.0001 (Fig. 3b); *F*_1,18_ = 671, *p* < 0.0001 (Fig. 3d) and *F*_1,18_ = 986, *p* < 0.0001 (Fig. 3f). The group factor was also significant for M100907, SB242084: *F*_1,18_ = 23, *p* < 0.0001; *F*_1,18_ = 421, *p* < 0.0001 and WAY100635 *F*_1,18_ = 23, *p* < 0.0001 (Fig. 3b, d and f, respectively). The group factor was significant for 25I-NBOMe + M100907 and 25I-NBOMe + SB242084: *F*_1,18_ = 28, *p* < 0.0001; *F*_1,18_ = 431, *p* < 0.0001, respectively, and for 25I-NBOMe + WAY100635: *F*_1,18_ = 28, *p* < 0.0001 (Fig. 3b, d and f, respectively). Post hoc Tukey’s test showed significant difference between control and 25I-NBOMe (1 and 3 mg/kg) and 25I-NBOMe + M100907, 25I-NBOMe + SB242084 and 25I-NBOMe + WAY100635 groups (*p* < 0.01) and between 25I-NBOMe + M100907 and 25I-NBOMe + SB242084 and 25I-NBOMe + WAY100635 in comparison to 25I-NBOMe alone (*p* < 0.01) (Fig. 2b, d and f).

### Basal levels of glutamate, DA and 5-HT in the rat frontal cortex

In the rat, basal levels of glutamate expressed in ng/10 μl of dialysate in the frontal cortex averaged 2.88 ± 0.18 (*n* = 48). Basal DA levels expressed in pg/10 μl of dialysate were 1.56 ± 0.09 (*n* = 48). 5-HT basal levels expressed in pg/10 μl of dialysate were 0.73 ± 0.05 (*n* = 48). No differences in basal levels between experimental groups were observed. The values were not corrected for the in vitro probe recovery.

### The effect of M100907, SB242084 and WAY100635 on 25I-NBOMe-induced wet dog shakes in rats

**Fig. 4:**
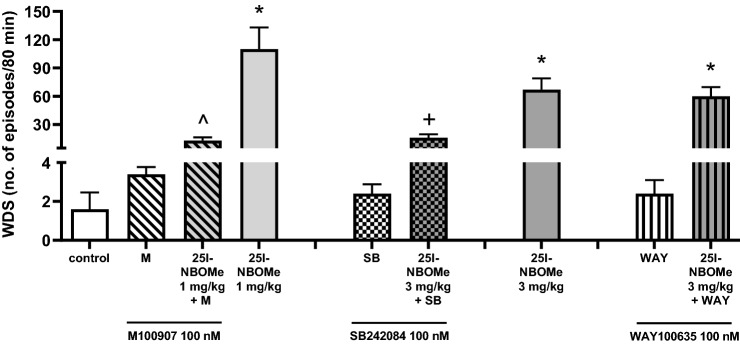
The effect of 25I-NBOMe and M100907, SB242084 and WAY100635, respectively on wet dog shakes (WDS). The number of WDS episodes was counted for 240 min starting immediately after the drug administration. Values are the mean ± standard error of the mean (SEM), *n* = 5–14 per experimental group. *p < 0.02 vs. control group; ^*p* < 0.05 25I-NBOMe vs. 25I-NBOMe + M100907; + *p* < 0.02 25I-NBOMe vs. 25I-NBOMe + SB242084 group (two-way ANOVA and Tukey’s post hoc test)

Rats treated with saline, M100907, SB242084 or WAY100635 (100 nM) showed a minimal behavior rated as WDS (Fig. 4). Injection of 25I-NBOMe-induced WDS, and the response after the dose of 1 mg/kg (*F*_1,23_ = 6.47, *p* < 0.02) was more potent than after 3 mg/kg (*F*_1,25_ = 6.57, *p* < 0.02) (Fig. 4). M100907 and SB242084 but not WAY100635 significantly suppressed 25I-NBOMe-induced WDS response (Fig. 4). Two-way ANOVA showed a significant effect of M100907 (*F*_1,23_ = 4.53, *p* < 0.05), SB242084 (*F*_1,25_ = 6.57, *p* < 0.02) but not WAY100635 (*F*_1,25_ = 0.09, *p* < 0.76).

## Discussion

Acute 25I-NBOMe treatment enhanced extracellular levels of glutamate, DA and 5-HT in the rat frontal cortex. Local intra-cortical administration of the selective serotonin 5-HT_2A_, 5-HT_2C_ and 5-HT_1A_ receptor antagonists M100907, SB242084 and WAY100635, respectively, at a concentration of 100 nM inhibited the increase in the DA and 5-HT levels, while the increase in the glutamate level was only reduced by M100907 and SB242084, but not by WAY100635. Furthermore, 25I-NBOMe-induced hallucinogenic activity in rats was attenuated by the 5-HT_2A_ and 5-HT_2C_ antagonists M100907 and SB242084 but not by WAY100635.

As shown in several studies, the 5-HT_2A_ receptor in the frontal cortex is critically involved in hallucinogenic activity in rodents [6, 14, 16, 19, 25]. Furthermore, the glutamatergic system also appears to play a role in the effects of serotonergic hallucinogens since in vivo studies have shown that the stimulation of cortical 5-HT_2A_ receptors caused the release of glutamate [6, 7, 9]. 25I-NBOMe is a potent 5-HT_2A_ receptor agonist with sub-nanomolar affinity and it is slightly weaker at 5-HT_2C_ receptors, while showing low μM affinity at 5-HT_1A_ receptors [1]. Our earlier study showed an inverted “U”-shaped dose–response effect of 25I-NBOMe in increasing extracellular DA and 5-HT levels and a “U”-shaped dose–response effect in glutamate release and hallucinogenic activity in rats [6]. Notably, the 25I-NBOMe dose of 1 mg/kg was most potent in increasing glutamate release and hallucinogenic activity, while the dose of 3 mg/kg was weakest [6]. We hypothesized that 25I-NBOMe at a dose of 1 mg/kg exerted its effect on WDS and glutamate release via the 5-HT_2A_ receptors, while at higher doses, the response was negatively modulated via the 5-HT_2C_ and 5-HT_1A_ receptors. Local perfusion of 100 nM M100907, a selective 5-HT_2A_ receptor antagonist significantly reduced WDS response and increased cortical glutamate release. Our data are consistent with the earlier studies indicating the reduction of the behavioral effect of 25I-NBOMe or DOI by M100907 in mice and rats [13, 16, 18]. In the aforementioned studies, the 5-HT_2A_ antagonist was administered peripherally, while in our experiments, M100907 was infused through a microdialysis probe directly into the frontal cortex. Assuming 8–10% in vitro probe recovery, the concentration of the antagonist in the close proximity of the probe was in low nM range, ensuring selective response. The effect of 25I-NBOMe at a higher dose of 3 mg/kg on the glutamate release and WDS was significantly decreased by local infusion of 100 nM SB242084, a 5-HT_2C_ selective receptor antagonist. As mentioned in Introduction, the 5-HT_2A_ receptors are mainly expressed on the apical dendrites of pyramidal cells in layer V of the cerebral cortex [3] with a minor localization to GABAergic interneurons [4, 5]. In contrast, the 5-HT_2C_ receptors are mainly expressed on GABAergic cells in the deep layers of the prefrontal cortex and may exert an inhibitory tone on pyramidal neurons by GABA release [10, 11]. Thus, the activation of 5-HT_2C_ receptors located on GABAergic interneurons by a higher dose of 25I-NBOMe could be the cause of a weaker effect of the compound on glutamate release and WDS response. Consequently, it may be expected that the 5-HT_2C_ receptor blockade by SB242084 should disinhibit pyramidal neurons by liberating them from inhibitory control of GABA, resulting in the increased release of glutamate and increased WDS response in comparison to 25I-NBOMe alone group. Unexpectedly, local infusion of SB242084 in the frontal cortex decreased 25I-NBOMe-induced glutamate release and WDS to the control values. This effect seems to be in contrast to the study by Vickers et al. [25] which demonstrated that a 5-HT_2C_ receptor agonist inhibited 5-HT_2A_ receptor-mediated head twitches in rats while the number of head-twitches induced by 5-HT_2A_ receptor agonists was greater in the presence of SB242084 [25]. Similarly, 5-HT_2C_ antagonists dose-dependently increased head-twitch behavior elicited by the 5-HT_2A_ agonist, DOI in mice [16]. On the other hand, the DOI-elicited head-twitch response was reduced in 5-HT_2C_ receptor-knockout mice [21] indicating that 5-HT_2C_ receptors are required for this behavior. Our contrasting data may be explained, to some extent, by a different way of drug administration. In the cited papers, 5-HT receptor antagonists were administered systematically, while in the present work, the drugs were infused locally into the frontal cortex. Thus, only a limited population of receptors could have been affected during infusion into the frontal cortex whereas receptors in other locations as fully functional might allow for adjustment of the response. Furthermore, co-expression of the 5-HT_2A_ and 5-HT_2C_ serotonin receptors on cells in the frontal cortex and their functional interaction needs to be taken into account for explanation of our data. The work of Nocjar et al. [10] demonstrated that a majority (73%) of cells that co-express the 5-HT_2C_ and 5-HT_2A_ receptors are GABA cells, and only some pyramidal cells (remaining 27%) were found to express the 5-HT_2C_ ad 5-HT_2A_ receptors in layers V–VI of the medial prefrontal cortex. However, only 28–53% of 5-HT_2A_ receptor-expressing pyramidal cells co-expressed 5-HT_2C_ receptor mRNA [26]. Hence, 5-HT_2C_ and 5-HT_2A_ receptor co-expression may affect cortical cell function in a different way. Taking into account differential location of receptor subtypes, 5-HT_2A_ receptor agonists enhance local pyramidal cell excitation [27], while local treatment with 5-HT_2C_ agonists triggers GABA cell excitation, but in turn, the released GABA may inhibit pyramidal cell function [28]. It has been suggested that GABAergic cells expressing both receptors are most likely regulated by their functional balance as evidenced at the level of intraneuronal signaling pathways. 5-HT_2A_ and 5-HT_2C_ receptors activate many of the same second messenger signaling systems [29, 30], but they may also recruit different intracellular signaling routes [31]. Data show that GABA cell stimulation induced by intra-cortical infusion of the 5-HT_2A/2C_ receptor agonist DOI was partially blocked by a 5-HT_2C_ antagonist, while being completely blocked by dual 5-HT_2A/2C_ antagonism [32]. Since 5-HT_2A_ and 5-HT_2C_ receptors are co-expressed on both pyramidal and GABAergic cells in the frontal cortex, thus, apart from 5-HT_2A_ receptors, 5-HT_2C_ receptors with pyramidal location may take part in the 25I-NBOMe-induced increase in extracellular glutamate level and this effect may be suppressed by local infusion of the 5-HT_2C_ receptor antagonist SB242084. Another possibility of pyramidal cell regulation could involve the blockade of 5-HT_2C_ receptors located on GABAergic cells. In the presence of SB242084, the activation of 5-HT_2A_ receptors on GABAergic interneurons may indirectly inhibit glutamate release from pyramidal cells by increasing GABA release.

Other serotonergic receptor subtypes are also co-expressed within the frontal cortex. 5-HT_1A_ and 5-HT_2A_ receptors are co-localized on the majority of pyramidal cells [17, 33] and there is evidence of their opposing cross-talk [17]. Thus, excitation of pyramidal neurons by 5-HT_2A_ receptor agonists might be reversed by inhibitory 5-HT_1A_ receptor agonists or potentiated by antagonists of this receptor. In light of this cross-talk, 5-HT_1A_ antagonists can augment the HTR induced by hallucinogen administration. However, infusion of the 5-HT_1A_ receptor antagonist WAY100635 did not change the 25I-NBOMe effect on the glutamate release and WDS response, as shown in our study. The help in answering this question may come from a recent report by Yuen et al. [34], who indicated that local 5-HT_2A/2C_ receptor co-activation opposed the function of inhibitory 5-HT_1A_ receptor. Possibly, co-administration of 25I-NBOMe as an agonist of 5-HT_2A/2C_ receptors with WAY100635 could alter the sensitivity of 5-HT_1A_ receptor and dampen its signaling pathways. Another issue which has to be considered to explain the observed effect is the receptor interaction profile of 25I-NBOMe. The in vitro affinity of 25I-NBOMe for 5-HT_1A_ receptors is ca. 2000 fold lower than for 5-HT_2A_ receptors [1]. Therefore, likely much higher concentration of 25I-NBOMe in the vicinity of 5-HT_1A_ receptors is necessary to counterbalance the agonist effect of this hallucinogen via 5-HT_1A_ receptors on the WDS response and glutamate release in the frontal cortex.

Frontal cortex neurons play an important role in the direct and indirect processing of information along the basal ganglia circuits via 5-HT receptors. Cortical output neurons innervate serotonergic dorsal and medial raphe cells and midbrain dopaminergic neurotransmission. In turn, the frontal cortex receives serotonergic and dopaminergic input from the midbrain raphe and ventral tegmental area (VTA) neurons [35, 36]. We observed lower extracellular DA and 5-HT levels in groups treated with 25I-NBOMe and M100907 or SB242084. It seems likely that weakened stimulation from the descending glutamatergic pathways onto raphe and VTA cells after co-administration of these drugs results in the reduced effect of the drugs on midbrain raphe and VTA neurons leading to lower DA and 5-HT release from the neuronal terminals in the frontal cortex. Surprisingly, in spite of no effect of WAY100635 on the 25I-NBOMe-induced glutamate release, lower extracellular DA and 5-HT levels were found in groups receiving this drug combination. It has been evidenced that the 5-HT_1A_ receptors are located postsynaptically on pyramidal and GABAergic neurons of the cortex [33, 37]. However, 5-HT_1A_ agonists were reported to act preferentially on GABAergic neurons [38]. Therefore, under conditions of 25I-NBOMe and WAY100635 co-administration, the blockade of 5-HT_1A_ receptors by WAY100635 may disinhibit GABA cells by releasing them from 5-HT_1A_ receptor control with subsequent decrease in output from DA and 5-HT terminals.

To conclude, the results of our study indicate that predominantly 5-HT_2A_ receptor is crucial in the regulation of glutamate, DA and 5-HT release from pyramidal cells and in hallucinogenic activity of 25I-NBOMe, however, 5-HT_2C_ subtype seems to be also required in the neurochemical and behavioral effects of this hallucinogen. The lack of noticeable influence of 5-HT_1A_ receptor blockade on the WDS response and glutamate release in the frontal cortex seems to result from a low binding affinity of 25I-NBOMe at these sites or damping of 5-HT_1A_ receptor signaling by 5-HT_2A/2C_ receptors. The suppressive effect of WAY100635 on DA and 5-HT release in the frontal cortex may result from disinhibition of GABA interneurons by 5-HT_1A_ receptor blockade.

## Electronic supplementary material

Below is the link to the electronic supplementary material.

Supplementary file1 (PDF 53 KB)
