# Supplementary material for: Contribution of serotonin receptor subtypes to hallucinogenic activity of 25I-NBOMe and to its effect on neurotransmission
Source: Pharmacol Rep. 2020 Nov 10;72(6):1593–603. doi: 10.1007/s43440-020-00181-4 (PMC7704505; doi:10.1007/s43440-020-00181-4)
Supplement: Supplementary file 1 — Supplementary file1 (PDF 53 KB) [file 43440_2020_181_MOESM1_ESM.pdf]

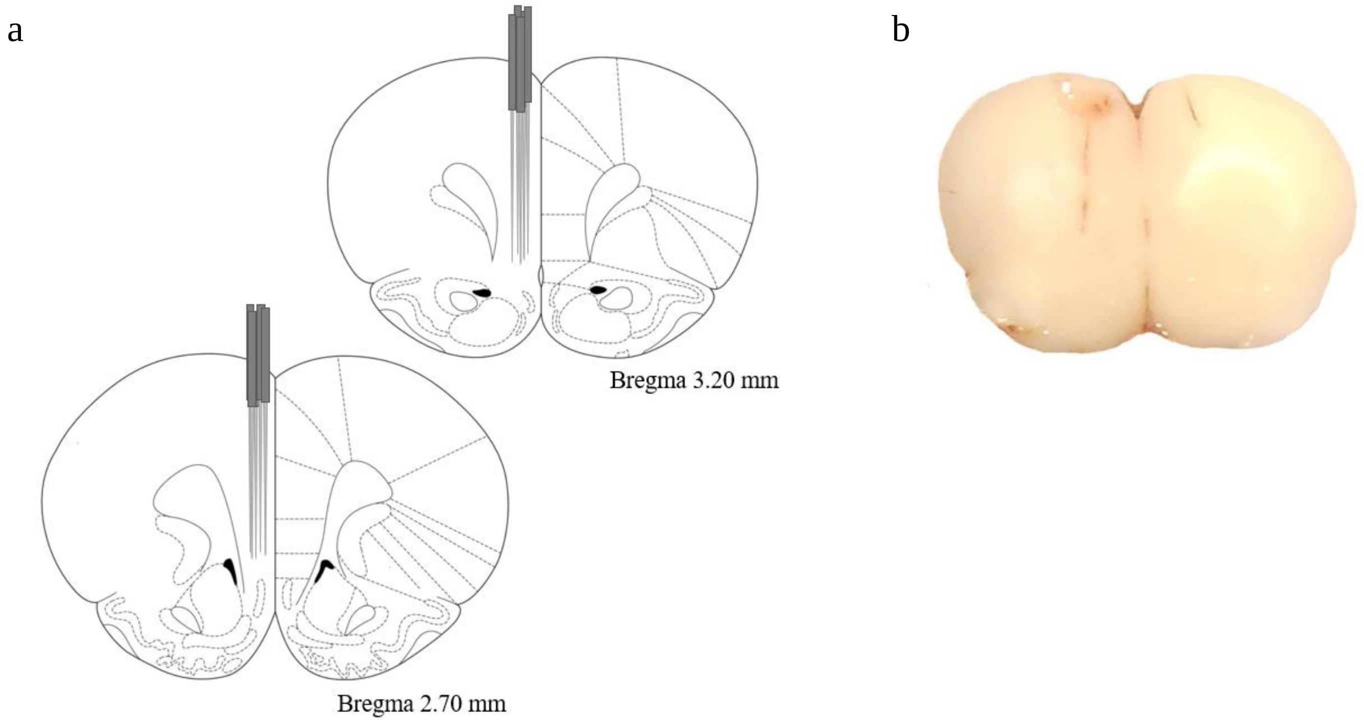

Fig. 1S. Microdialysis probe placements: (a) schematic diagram of representative probe placements in the rat frontal cortex and (b) photomicrograph illustrating the histological verification of typical probe placement.
